# Decreased Semaphorin3A expression correlates with disease activity and histological features of rheumatoid arthritis

**DOI:** 10.1186/1471-2474-14-40

**Published:** 2013-01-23

**Authors:** Shu Takagawa, Fumio Nakamura, Ken Kumagai, Yoji Nagashima, Yoshio Goshima, Tomoyuki Saito

**Affiliations:** 1 Department of Orthopaedic Surgery, Yokohama City University Graduate School of Medicine, 3-9 Fukuura, Kanazawa-ku, Yokohama 236-0004, Japan; 2 Department of Molecular Pharmacology and Neurobiology, Yokohama City University Graduate School of Medicine, 3-9 Fukuura, Kanazawa-ku, Yokohama 236-0004, Japan; 3 Department of Molecular Pathology, Yokohama City University Graduate School of Medicine, 3-9 Fukuura, Kanazawa-ku, Yokohama 236-0004, Japan

**Keywords:** Rheumatoid arthritis, Semaphorin3A, Disease activity score, Histological scoring

## Abstract

**Background:**

Rheumatoid arthritis (RA) is an autoimmune disease of which the pathogenetic mechanisms are not fully understood. Semaphorin3A (Sema3A) has an immune regulatory role. Neuropilin1 (NRP1), the primary receptor for Sema3A, is also a receptor for vascular endothelial growth factor 165 (VEGF_165_). It has been shown that Sema3A competitively antagonizes VEGF165 signaling. This study investigated whether Sema3A is expressed in synovial tissues, and is associated with disease activity and the histological features of synovial tissues from RA patients.

**Methods:**

Human synovial tissues samples were obtained from RA and osteoarthritis (OA) patients. Disease activity of RA patients was calculated using the 28-joint Disease Activity Score based on C-reactive protein (DAS28-CRP). The histological features of RA synovial tissues were evaluated using Rooney’s inflammation scoring system. The localization of Sema3A, VEGF_165 _and NRP1 positive cells was immunohistochemically determined in synovial tissues. Expression levels of *Sema3A, VEGF-A* and *NRP1* mRNA were determined using quantitative real-time polymerase chain reaction (qPCR).

**Results:**

In OA specimens, Sema3A, VEGF_165 _and NRP1 proteins were expressed in the synovial lining and inflammatory cells beneath the lining. Immunohistochemistry revealed the protein expression of Sema3A in synovial lining cells was decreased in RA tissues compared with OA samples. qPCR analysis demonstrated a significant reduction of *Sema3A* mRNA levels in RA synovial tissue samples than in OA and a significant correlation of the ratio of *Sema3A*/*VEGF-A* mRNA expression levels with DAS28-CRP (R = −0.449, p = 0.013). *Sema3A* mRNA levels also correlated with Rooney’s inflammation score, especially in perivascular infiltrates of lymphocytes (R = −0.506, p = 0.004), focal aggregates of lymphocytes (R = −0.501, p = 0.005) and diffuse infiltrates of lymphocytes (R = −0.536, p = 0.002).

**Conclusions:**

Reduction of Sema3A expression in RA synovial tissues may contribute to pathogenesis of RA.

## Background

Rheumatoid arthritis (RA) is a chronic inflammatory disease characterized by progressive joint destruction that accompanies the proliferation of synovial cells and blood vessels as well as invasion of inflammatory cells [1-3]. Although the initiating factors of RA are unknown, autoimmune reactions are activated in connective tissues. In RA joints, immune cells such as T and B cells invade the hyperplastic synovial membranes [4]. Activated synovial T and B cells secrete various types of pro-inflammatory cytokines including interleukin-1 (IL-1), IL-17 and tumor necrosis factor-α (TNF-α). These cytokines induce the synthesis of matrix degrading enzymes in chondrocytes. Synovial fibroblasts also produce matrix-degrading enzymes and can invade cartilage, leading to its destruction [3,4].

Semaphorins are a large family of proteins that function as guidance cues for axonal/dendritic projections. Class 3 semaphorins are vertebrate secreted proteins and include seven members, semaphorin3A (Sema3A) to Semaphorin3G (Sema3G) [5]. Sema3A is a repulsive factor for sensory fibers, and Semaphorin3C (Sema3C) and Semaphorin3F (Sema3F) repulse sympathetic nerve fibers [6]. Whereas the primary receptor for Sema3A is neuropilin1 (NRP1), Sema3F has a higher binding affinity to neuropilin2 (NRP2). Sema3C binds to both NRP1 and NRP2 [7,8].

The action of Sema3A is not limited to the nervous system as NRP1 is expressed on endothelial cells, keratinocytes, T cells, and tumor cells in breast and prostate cancer. Sema3A inhibits angiogenesis, migration of keratinocytes, proliferation of T cells, and migration of tumor cells [8-11]. In addition, it was recently shown that Sema3A is involved in the entry of dendritic cells to the lymphatic system [12]. Several studies have indicated that a reduction of Sema3A expression is involved in the exacerbation of autoimmune diseases, such as RA and systemic lupus erythematosus (SLE) [13,14].

NRP1 mediates signal transduction through PlexinA co-receptors [15], which are classified into four sub-families, PlexinA1–4 [16]. The Sema3A/NRP1/PlexinA complex regulates the actin cytoskeleton through small G-proteins, including Rac and Rho [17]. In immune cells, the Rac family is associated with the proliferation and activation of B cells [18], and the activation of T cells induced by dendritic cells [19]. NRP1 is also a putative marker of regulatory T cells [20], and therefore Sema3A/NRP1/PlexinA signaling may modulate regulatory T cell functions.

Vascular endothelial growth factor165 (VEGF_165_), a spliced isoform of VEGF-A [21], binds to NRP1 [22]. VEGF is a key regulator of angiogenesis and is involved in the development of inflammation [23]. In RA patients, serum VEGF levels positively correlate with disease activity score and joint destruction [24,25]. Because NRP1 is a common receptor for Sema3A and VEGF_165 _[11,26], the efficacy of VEGF_165 _is attributed to Sema3A expression. Indeed, the imbalance between Sema3A and VEGF expression levels is also associated with disease activity in several tumors [27-29].

These facts prompted us to investigate the possibility that Sema3A expression and/or the balance of Sema3A and VEGF_165 _expression may regulate the disease activity of RA including inflammation, angiogenesis and proliferation of synovial cells. We found that Sema3A expression was decreased in RA synovial tissues when compared with osteoarthritis (OA) samples. The Sema3A expression level was also significantly associated with the RA pathological score and disease activity score.

## Methods

### Patients and samples

Synovial tissue samples were obtained from RA (n = 30) and OA (n = 23) patients during total knee arthroplasty. The diagnosis of patients with RA and OA was based on the revised 1987 American Rheumatism Association Criteria for RA [30] and the American Rheumatism Association Criteria for OA [31], respectively, as shown in Table 1. Before arthroplasty, the disease activity of each RA patient was evaluated using the 28-joint Disease Activity Score based on C-reactive protein (DAS28-CRP) [30,32]. This study was approved by the Ethics Committee of Yokohama City University Graduate School of Medicine, and written informed consent was obtained from all patients involved in this study (notice of approval Institutional Review Board protocol number: B1100513031).

**Table 1:** Characteristics of patients with OA and RA

	**OA (n = 23)**	**RA (n = 30)**
Male/female, n	2/21	6/24
Age, mean ± SD (range, years)	76.6 ± 5.8 (64–84)	69.4 ± 8.1 (55–83)
Disease duration, mean ± SD (years)	10.7 ± 5.1	16.3 ± 9.1
CRP, mean ± SD (mg/dl)	0.16 ± 0.30	1.43 ± 1.21
DAS28-CRP, mean ± SD	**–**	3.74 ± 0.84
**Medications**		
Prednisolone, n (%)	NA	17 (56.7)
Methotrexate, n (%)	NA	15 (50.0)
Sulfasalazine, n (%)	NA	7 (23.3)
Bucillamine, n (%)	NA	6 (20.0)
Cyclosporine, n (%)	NA	3 (10.0)
Actarit, n (%)	NA	1 (3.3)
TNF-α blockade, n (%)	NA	4 (13.3)

n, number; OA, osteoarthritis; RA, rheumatoid arthritis; SD, standard deviation; CRP, C-reactive protein; DAS28, 28-joint Disease Activity Score; NA, not applicable.

### Hematoxylin and eosin (HE) staining for histological assessment

Tissue specimens were fixed with 20% formalin and embedded in paraffin. Paraffin sections (4 μm thick) were stained with HE. The histological features of RA synovial tissues were evaluated using Rooney’s inflammation scoring system [33]. These histological parameters included the degree of synovial hyperplasia, fibrosis, the number of blood vessels, focal and diffuse aggregates of lymphocytes and perivascular infiltrates of lymphocytes. All parameters were scored separately on a scale of 0–10 (Table 2). Two investigators (S.T. and Y.N.) independently assessed the histologic severity.

**Table 2:** Rooney’s inflammation scoring system for patients with rheumatoid arthritis

	**Score**										
**Histologic feature**	**0**	**1**	**2**	**3**	**4**	**5**	**6**	**7**	**8**	**9**	**10**
Synoviocyte hyperplasia^a^	1	2	3	4	5	6	7	8	9	10	>10
Fibrosis^b^	<10	<15	<20	<25	<30	<40	<50	<60	<70	<80	≥80
Proliferating blood vessels^c^	0–3	4–5	6–7	8–9	10–11	12–13	14–15	16–17	18–19	20–22	>22
Perivascular infiltrates of lymphocytes^d^	<5	10	20	30	40	50	60	70	80	90	100
Focal aggregates of lymphocytes^e^	<11	15	20	25	30	35	40	45	50	55	>55
Diffuse infiltrates of lymphocytes^f^	0	10	20	30	40	50	60	70	80	90	100

^a^A normal synoviocyte monolayer was scored as 0. The score was increased as the synoviocyte lining layer increased in depth. ^b^Sections containing < 10% fibrous tissue in the sublining layers were scored as 0. The score increased as the percentage of fibrosis in the section increased. ^c^Three or fewer vessels per high power field (HPF) were scored as 0. The score increased as the number of vessels per HPF increased. ^d^When no lymphocytes were observed around vessels, it was scored as 0. The score increased as the percentage of vessels surrounded by lymphocytes increased. Subsequently, the diameter of the perivascular lymphocytes was graded as mild = 2–4 cells in diameter, moderate = 5–7 cells in diameter, and severe = 8–10 cells in diameter. Where the perivascular lymphocytes were considered mild or severe, the original score was lowered by 1 point or raised by 1 point, respectively. ^e^Absence of focal aggregates of lymphocytes, which exceeded 10 cells in diameter, were scored as 0. The score increased as the cell numbers in the diameter of the focal aggregates increased. ^f^An estimate was made of the percentage of cells per HPF that were lymphocytes. The score increased as the percentage of lymphocytes increased.

### Immunohistochemistry

For immunostaining of Sema3A, antigen retrieval was performed by incubating at 95°C for 30 min in DAKO Target Retrieval Solution (pH 9.0; DAKO, Glostrup, Denmark). For immunostaining of NRP1, VEGF_165_, CD3 and CD20, the tissue sections were subjected to antigen retrieval by autoclaving in 10 mM citrate buffer (pH 6.0) for 15 min at 121°C. Slides were then treated with 0.3% H_2_O_2 _for 30 min to block endogenous peroxidases. Sections for Sema3A, NRP1 and VEGF_165 _staining were blocked with 10% normal goat serum. All sections were incubated at 4°C overnight with rabbit anti-Sema3A polyclonal antibody (1:200; Abcam, Cambridge, UK), mouse anti-CD3 monoclonal antibody (1:50; DAKO), mouse anti-CD20 monoclonal antibody (DAKO), rabbit anti-NRP1 polyclonal antibody (1:100; Santa Cruz Biotechnology, Santa Cruz, CA, USA) or rabbit anti-VEGF_165 _polyclonal antibody (1:100; Millipore, MA, USA). This was followed by incubation with Envision™, Rabbit/HRP (DAKO) for Sema3A, NRP1 and VEGF_165 _or Envision™/HRP (DAKO) for CD3 and CD20. Immunoreactivity was visualized using 3,3’-diaminobenzidine plus (DAB+, DAKO) for Sema3A, NRP1 and VEGF_165_, or 3,3’-diaminobenzidine tetrahydrochloride (DAB; Sigma-Aldrich, St. Louis, MO, USA) for CD3 and CD20. Finally, the sections were counterstained with hematoxylin and mounted. Controls for Sema3A immunohistochemistry included preabsorption and co-incubation of the antibody with the antigen peptide (1 μl/ml; Abcam).

### Quantification of Sema3A immunostaining

Tissue sections immunostained with anti-Sema3A antibody were analyzed for 12 patients with OA and 12 patients with RA. The total immunostaining intensity in the lining layer was measured using a BZ-9000 microscope (Keyence, Osaka, Japan) equipped with Dynamic Cell count software BZ-H1C (Keyence). Immunostaining intensity per unit was calculated as described previously [34].

### Quantitative real-time polymerase chain reaction (qPCR)

Total RNA was extracted from synovial tissues using an Illustra RNA spin Mini Kit (GE Healthcare, Buckinghamshire, UK) according to the manufacturer’s instructions. RNA was reversed transcribed into cDNA using a PrimeScript RT Reagent Kit (Takara Bio, Ohtsu, Japan). The cDNA synthesized from 1 μg of total RNA was used as the template in each reaction. qPCR analysis was performed using an Applied Biosystems 7900HT Fast Real-time PCR system (Applied Biosystems LLC) based on the TaqMan® PCR manufacturer’s protocol. The assay was performed in triplicate in optical 96-well reaction plates covered with optical adhesive cover in a volume of 10 μl containing 0.5 μl Taqman Gene Expression Assay 20X for human *Sema3A* (assay ID Hs01085496_m1, GenBank accession number NM_006080, Applied Biosystems LLC), *VEGF-A* (assay ID Hs00173626_m1, GenBank accession number NG_008732, Applied Biosystems LLC), *NRP1* (assay ID Hs00826128_m1, GenBank accession number NM_003873; Applied Biosystems LLC) and *β-actin* (assay ID 4326315E, GenBank accession number NM_001101; Applied Biosystems LLC), 5 μl Taqman Fast Advanced Master Mix 2X, 2 μl cDNA template and 2.5 μl RNase-free water. The default ABI 7900HT amplification conditions were 20 sec at 95°C, followed by 1 sec at 95°C and 20 sec at 60°C for 40 cycles. A standard curve, derived from known serial dilutions of RA synovial tissue, was constructed to calculate arbitrary values of mRNA levels and to correct for differences in primer efficiencies. The obtained data were standardized using the reference gene, *β-actin.*

### Double-staining Immunofluorescence

Synovial tissue specimens embedded in Optimum Cutting Temperature compound (Sakura Finetek Japan, Tokyo, Japan) were sectioned (5 μm thick). The sections were then fixed in cold acetone for 5 min at 4°C and rinsed in phosphate buffered saline (PBS). To eliminate nonspecific protein binding, the samples were incubated with 10% normal goat serum for 30 min at room temperature. The samples were incubated with rabbit anti-NRP1 polyclonal antibody (1:100; Santa Cruz Biotechnology) and mouse anti-CD20 monoclonal antibody (DAKO) overnight at 4°C. This was followed by incubation with Alexa 488-labeled goat anti-rabbit antibody (Applied Biosystems LLC, Foster City, CA, USA) and Alexa 594-labeled goat anti-mouse antibody (Applied Biosystems LLC) for 40 min at 37°C. Finally, the sections were mounted with aqueous mounting medium. The distributions were analyzed by confocal microscopy using a Zeiss LSM510 confocal laser microscope (Carl Zeiss, Oberkochen, Germany).

### Statistical analysis

Statistical analyses were performed using SPSS 11.0 for Windows (SPSS Inc, Chicago, IL). The Mann–Whitney *U*-test and Spearman’s rank correlation coefficient were used to test the differences. A p value < 0.05 was considered significant. We calculated a posterior power of this study using G*Power (Faul, Erdfelder, Lang, & Buchner, 2007). All statistical powers in this study were greater than 80%.

## Results

### Sema3A, VEGF_165_, NRP1 and CD3 expression in OA and RA synovial tissues

**Figure 1:**
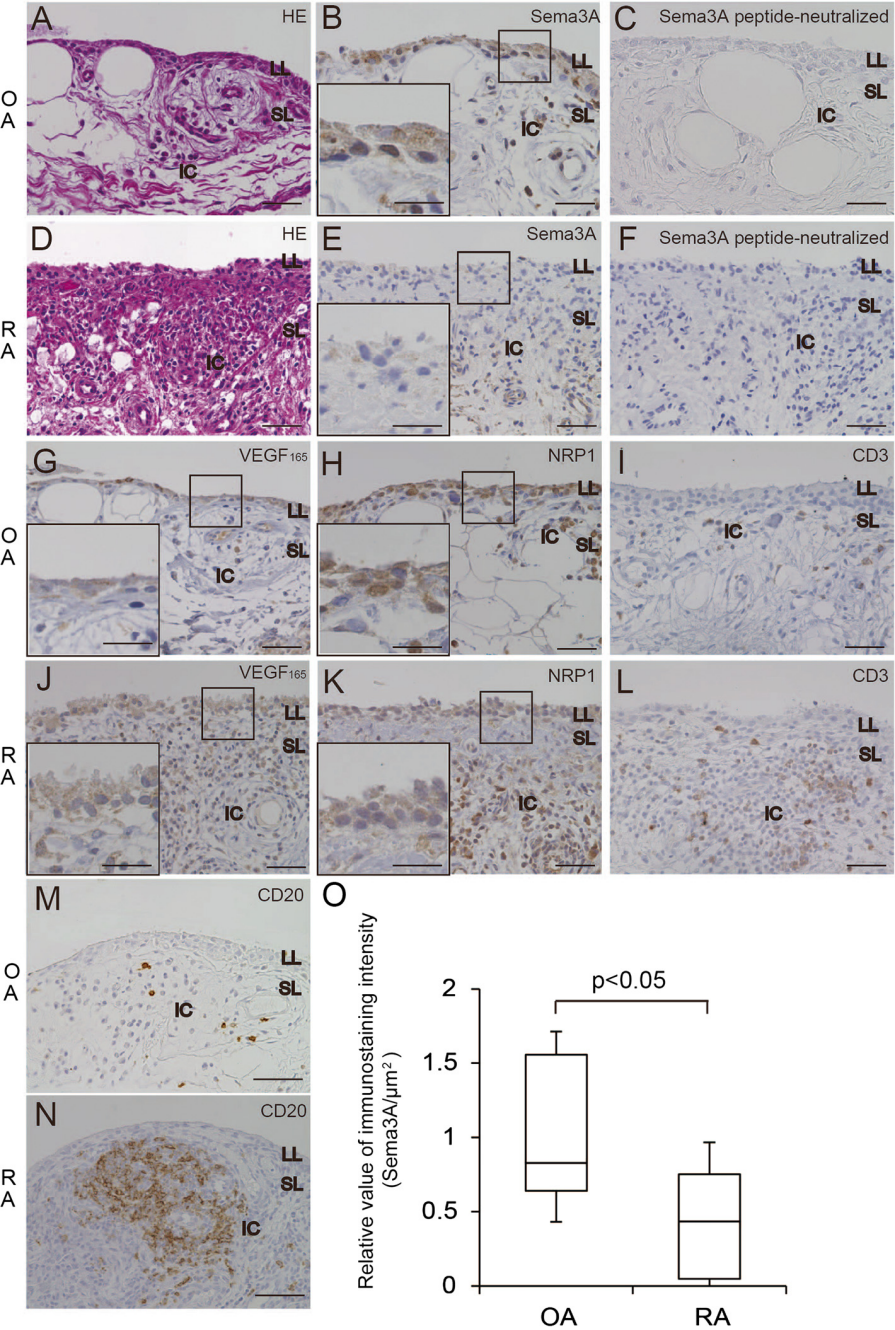
**Histological/immunohistochemical analysis of Sema3A, NRP1, VEGF**_**165 **_**and CD3 in OA and RA synovial tissue.** Representative HE staining of OA (**A**) and RA (**D**) synovial tissues. OA synovial tissues contain lining (LL) and sublining (SL) layers. RA synovial tissues are marked by the hyperplasia of synovial tissues in the lining layer and numerous infiltrated inflammatory cells (IC) in the sublining layer. Sema3A expression was detected in the lining layer and inflammatory cells in the sublining layer of OA (**B**) and RA (**E**) synovial tissues. The density of the Sema3A signal in the lining layer was lower in RA than OA. Peptide-neutralized anti-Sema3A antibodies did not stain tissue sections from OA (**C**) and RA (**F**). NRP1 and VEGF_165 _localized to the same areas as Sema3A in OA. VEGF_165_ expression in the lining layer in RA tissues was similar with OA (**G, J**). The NRP1 expression level in the lining layer of RA was similar to OA (**H, K**). T cells (CD3) and B cells (CD20) were detected among inflammatory cells in the sublining layer of OA and RA synovial tissues (**I, L, M, N**). The numbers of T cells and B cells were higher in RA synovial tissues compared with OA. Sections were counterstained with hematoxylin. Scale bars = 50 μm in the whole image view and 25 μm in the magnified view. Immunostaining of lining layer Sema3A was significantly decreased in RA (n = 12) synovial tissues compared with OA (n = 12) subjects (**O**). Results are presented as relative values compared with OA subjects. The box plots demonstrate the 10th and 90th percentile (whiskers), the 25th and 75th percentile, and the median. P values were obtained using the Mann–Whitney *U*-test.

To investigate the involvement of Sema3A in RA pathogenesis, we performed immunohistochemical staining for Sema3A, VEGF_165 _and NRP1 expression in serial synovial serial sections from RA and OA patients. HE staining demonstrated that synovial tissues from OA patients contained two layers, the lining and sublining layers (Figure 1A). Sema3A was mainly expressed in the lining layer and a small number of inflammatory cells were present in the sublining layer of OA samples (Figure 1B). This immunoreactive signal was abolished by the preincubation of anti-Sema3A antibody with the antigen peptide (Figure 1C), confirming the specificity of Sema3A immunostaining. In RA specimens, there was a marked increase in synovial tissue thickness of the lining layer caused by hyperplasia of synovial cells and numerous infiltrating inflammatory cells in the sublining layer (Figure 1D). The immunostaining signal of Sema3A in the hyperplastic lining layer was lower in RA tissues compared with OA samples (Figure 1E). Additionally, numerous inflammatory cells in the sublining layer expressed Sema3A. In OA and RA synovial tissues, VEGF_165_ was expressed in the lining layer and in the inflammatory cells of the sublining layer (Figure 1G, J). The VEGF_165_ expression density in the lining layer did not differ between RA and OA samples. NRP1, the shared receptor for Sema3A and VEGF_165_, was expressed in synovial cells of the lining layer, along with inflammatory cells and vascular endothelial cells in the sublining layer. The NRP1 expression density in the lining layer of RA was not significantly different to that of OA (Figure 1H, K). The localization of Sema3A, VEGF_165_, and NRP1 almost overlapped, suggesting that functional competition for Sema3A and VEGF_165 _may influence these cells. To evaluate the infiltration of immune cells, sections were stained with anti-CD3 antibody, a marker for T cells and anti-CD20 antibody, a marker for B cells. While T cells (CD3) were sporadically localized in the sublining layer of OA specimens, the number of T cells was greater in RA synovial tissues (Figure 1I, L). B cells were also sporadically localized in the sublining layer of OA specimens and large numbers of B cells were observed in the lymphoid follicles of RA synovial tissues (Figure 1M, N). We quantified Sema3A-immunostaining signal in the lining layer of RA and OA specimens. Sema3A-immunostaining intensity per unit area of lining layer was significantly less in RA patients compared with OA (Figure 1O).

### Expression of *Sema3A*, *VEGF-A* and *NRP1* mRNA and correlation with DAS28-CRP in RA

**Figure 2:**
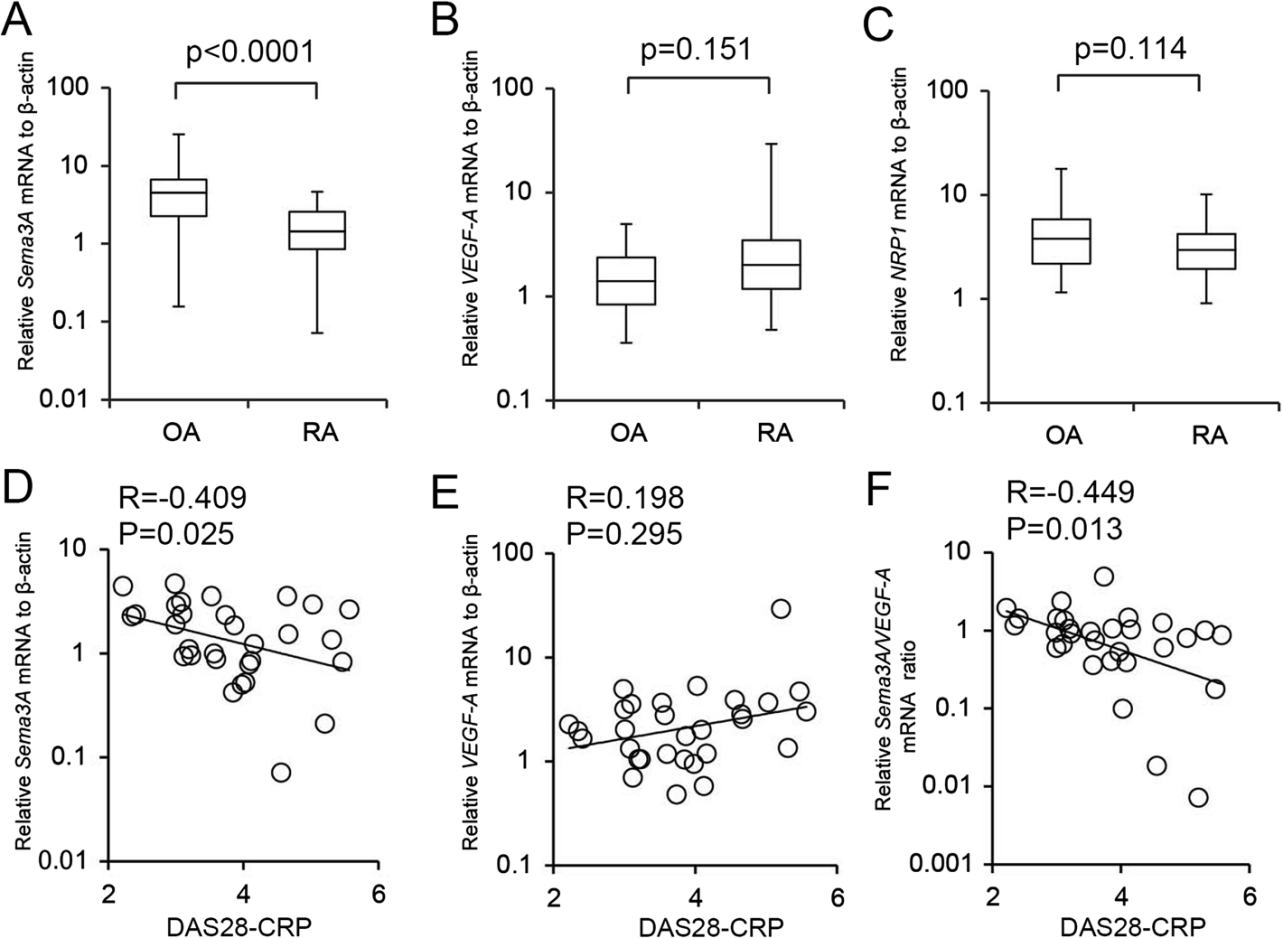
**Expression of *Sema3A, VEGF-A *and *NRP1 *mRNA in synovial tissues.** Expression levels of *Sema3A*, *VEGF-A* and *NRP1* mRNA were measured using real-time PCR. The mRNA levels were normalized to the expression of *β-actin*. The box plots demonstrate the 10th and 90th percentile (whiskers), the 25th and 75th percentile, and the median. *Sema3A* mRNA levels were significantly decreased in RA (n = 30) synovial tissues compared with OA (n = 23) (**A**). *VEGF-A* and *NRP1* mRNA levels were not significantly altered (**B, C**). Correlation of *Sema3A* mRNA levels and DAS28-CRP (**D**); *VEGF-A* mRNA levels and DAS28-CRP (**E**); and *Sema3A*/*VEGF-A* mRNA levels and DAS28-CRP (**F**). P values were obtained using the Mann–Whitney *U*-test. were examined using Spearman’s rank correlation coefficient.

To confirm changes in Sema3A expression in RA, we examined mRNA expression levels in OA and RA joint specimens using qPCR analysis. To evaluate the expression levels of *VEGF*_*165 *_we used *VEGF-A* primers in the qPCR experiments. *Sema3A* mRNA levels in synovial tissue samples were significantly lower in RA (mean expression level 1.80) than in OA (mean expression level 6.68; p < 0.0001; Figure 2A). The mRNA expression of *VEGF-A* or *NRP1* was not significantly different between RA and OA (Figure 2B, C). We also obtained similar results with VEGF_165_ primer in preliminary study (Additional file 1: Figure S1A). We next examined the correlation of *Sema3A* expression levels with DAS28-CRP, the disease activity score for RA. A negative correlation was observed between *Sema3A* expression levels and DAS28-CRP (R = −0.409, p = 0.025; Figure 2D). This suggested that the reduction of *Sema3A* expression might augment the disease activity of RA. *VEGF-A* expression levels did not significantly correlate with DAS28-CRP (R = 0.198, p = 0.295; Figure 2E). We also found a negative correlation between *Sema3A/VEGF-A* ratios and DAS28-CRP (R = −0.449, p = 0.013; Figure 2F).

### Correlation of *Sema3A* mRNA levels with histological features in RA synovial tissues

**Figure 3:**
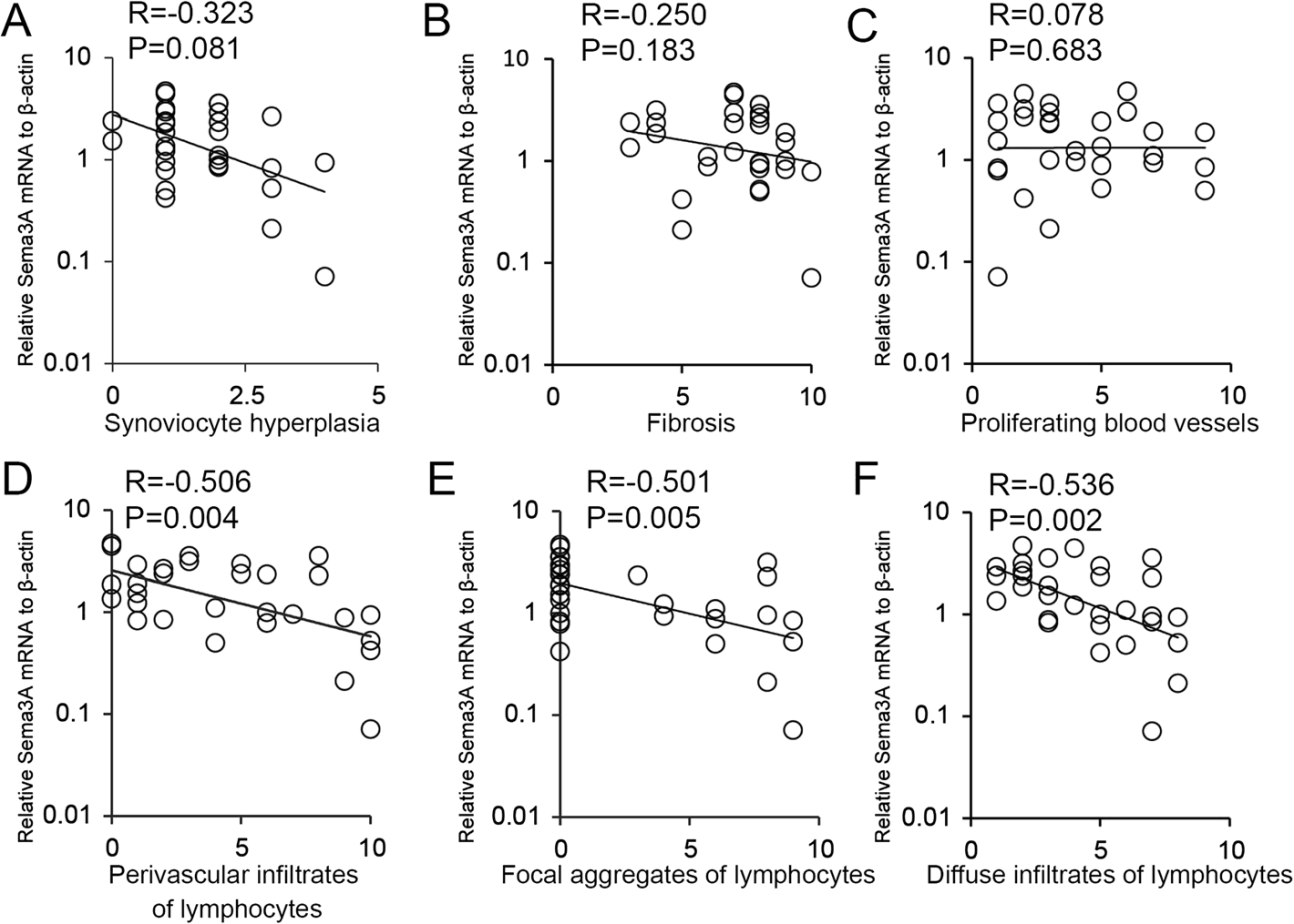
**Expression levels of *Sema3A *mRNA correlate with histological features in RA synovial tissues.** Correlations between *Sema3A* mRNA expression levels and histological parameters of RA synovial tissues were assessed. Histological parameters were scored separately on a scale of 0–10 according to Rooney’s inflammation scoring system. Correlation of *Sema3A* mRNA levels and synoviocyte hyperplasia (**A**); *Sema3A* mRNA levels and fibrosis (**B**); *Sema3A* mRNA levels and proliferating blood vessels (**C**); *Sema3A* mRNA levels and perivascular infiltrates of lymphocytes (**D**); *Sema3A* mRNA levels and focal aggregates of lymphocytes (**E**); and *Sema3A* mRNA levels and diffuse infiltrates of lymphocytes (**F**). Correlations were examined using Spearman’s rank correlation coefficient.

To confirm the inverse correlation of *Sema3A* mRNA expression levels and RA disease activity, we compared the relative *Sema3A* mRNA expression levels with histological parameters of RA synovial tissues. This included the degree of synovial hyperplasia, fibrosis, number of blood vessels present, perivascular infiltrates of lymphocytes, focal aggregates of lymphocytes and diffuse infiltrates of lymphocytes. No significant correlation between *Sema3A* expression and synovial hyperplasia, fibrosis or the number of blood vessels was observed (Figure 3A–C). In contrast, perivascular infiltrates of lymphocytes, focal aggregates of lymphocytes and diffuse infiltrates of lymphocytes significantly correlated with *Sema3A* mRNA expression levels (R = −0.506, p = 0.004; R = −0.501, p = 0.005; R = −0.536, p=0.002 respectively; Figure 3D–F). These results indicated that *Sema3A* mRNA reduction was mainly associated with immune reactions in the synovial tissue from RA patients.

### Expression of NRP1 in CD20-positive B cells in lymphocyte aggregates

**Figure 4:**
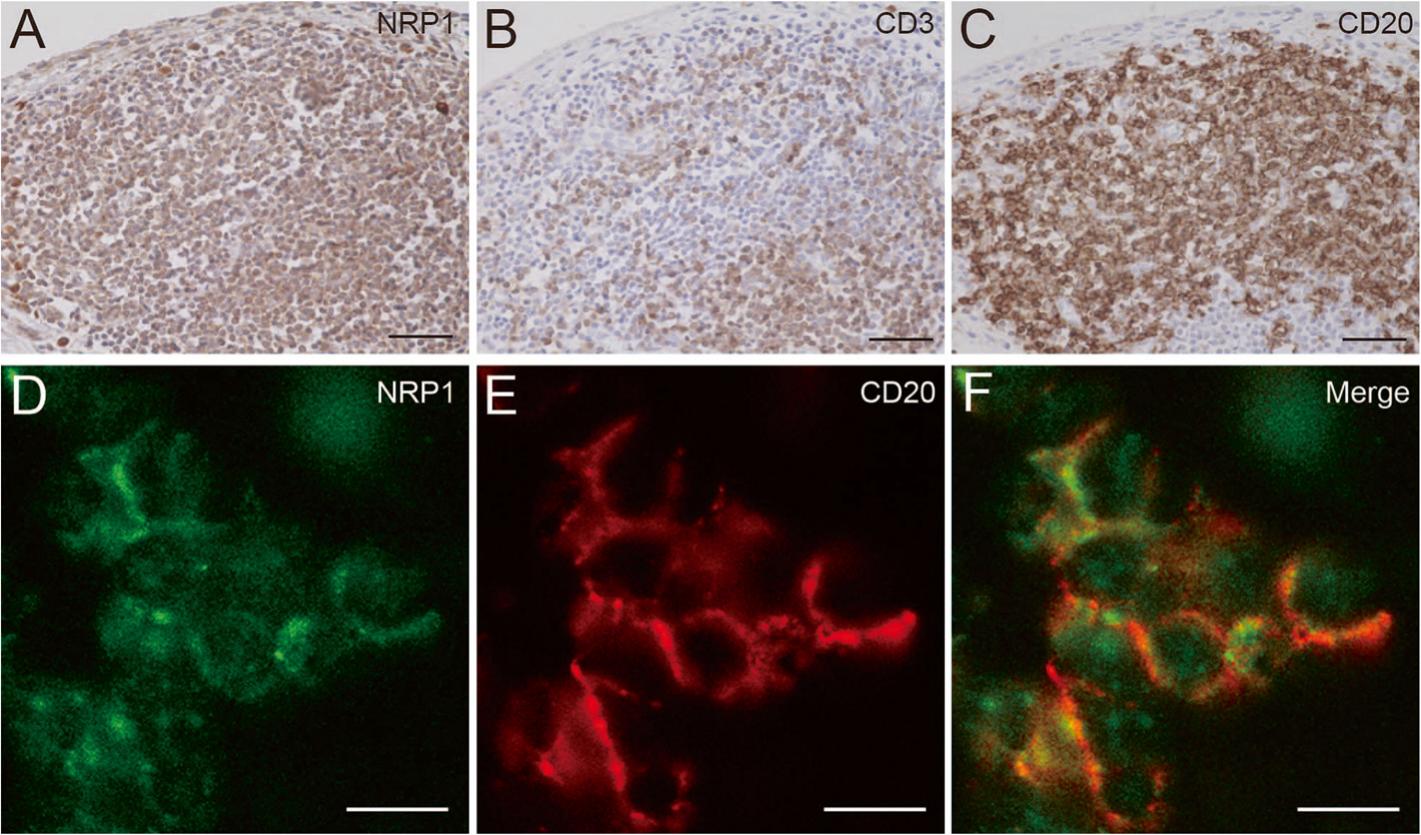
**Immunohistochemical and double immunofluorescence staining of lymphocyte aggregates.** NRP1 was abundantly expressed by inflammatory cells of lymphocytes aggregates (**A**). Immunohistochemical staining of sequential sections showed that NRP1-positive cells in lymphocytes aggregates also expressed CD3 (**B**) or CD20 (**C**). Micrographs show the double immunofluorescence detection of NRP1 (**D**, green) and CD20 (**E**, red) with the merged images (**F**). The NRP1 positive signal colocalized with the CD20 positive signal in the inflammatory cells of lymphocyte aggregates. A–C, scale bars = 50 μm; D–F, scale bars = 10 μm.

Histological scores and *Sema3A* expression levels revealed that a decrease in Sema3A augmented focal aggregates of lymphocytes. To examine the cell types of NRP1-positive inflammatory cells, we stained sequential tissue sections with markers for T cells (CD3) and B cells (CD20). NRP1 was abundantly expressed in the inflammatory cells of lymphoid follicles (Figure 4A). In RA synovium, T cells, B cells and dendritic cells [35] can be arranged in sophisticated organizations that resemble the microstructures usually formed in secondary lymphoid organs. Interestingly, NRP1-expressing dendritic cells also exist in the lymphoid follicle/lymphocyte focal aggregates. Immunohistochemical staining of sequential slices from the lymphoid follicles showed that NRP1-positive cells also expressed CD3 or CD20 (Figure 4B, C). Since lymphoid follicles were mainly composed of B cells [36], we also performed double immunofluorescence staining of NRP1 and CD20. The NRP1 positive signal colocalized with the CD20 positive signal in the inflammatory cells of lymphocyte aggregates (Figure 4D-F).

## Discussion

It has been suggested that abnormalities of the immune system play an important role in the pathogenesis of chronic inflammatory conditions such as RA. This study revealed *Sema3A* expression levels significantly correlated with the histological score of perivascular infiltrates of lymphocytes, focal aggregates of lymphocytes and diffuse infiltrates of lymphocytes. Sema3A is involved in immune responses, and its expression levels are altered in several autoimmune diseases [29,37,38]. The NRP1 and PlexinA complex mediates Sema3A signaling in the immune system [39]. NRP1 is expressed by regulatory T cells, a subset of T cells [40] and PlexinA4 regulates T cell proliferation and activation through inhibition of the actin-cytoskeleton rearrangement and T cell receptor polarization [9]. Recently, it was reported that Sema3A promotes regulatory T cells by enhancing IL-10 production [14]. Regulatory T cells play an important role in maintaining immunological self-tolerance by suppressing autoreactive T cells [41]. Immunohistochemical staining in the current study revealed that Sema3A is expressed in the lining layer. Thus, in OA joints, secreted Sema3A from the lining layer may enhance regulatory T cell functions to suppress autoimmune responses in the sublining layer. In RA, the reduction of Sema3A may abrogate the functions of regulatory T cells, thus allowing the infiltration and focal aggregation of autoreactive lymphocytes in the sublining layer. Further investigation of the correlation between Sema3A and IL-10 expression levels may increase our understanding of their roles in RA.

Catalano reported synovial tissues derived from healthy controls, OA and RA patients exhibited no significant differences although the relative Sema3A expression was lowest in RA samples [14]. This discrepancy might be explained by the difference in sampling numbers, which can influence statistical power, and/or by different qPCR methods used. We quantified *Sema3A* mRNA expression in a larger number of RA patients (n = 30) and OA patients (n = 23), whereas Catalano used relatively small numbers of RA (n = 10), OA (n = 10) patients and healthy controls (n = 5). For qPCR, we used the standard curve method for relative quantification, whereas Catalano used a comparative Ct method.

Sequential immunohistochemical staining revealed that B cells were observed mainly in the lymphoid follicles, consistent with a previous report [36]. Here we observed that B cells in the follicles expressed NRP1 (Figure 4). Vadasz *et al.* recently suggested that Sema3A can modulate the autoimmune properties of B cells in SLE [13]. Thus, Sema3A may exert similar functions in NRP1-positive B cells from RA synovial tissues. Vadasz *et al.* also reported Sema3A serum levels were significantly lower in SLE patients (p < 0.0001) and RA patients (p = 0.047) compared with healthy controls [13]. Since Sema3A is a secreted soluble protein and can enter the systemic circulation [16,42], lower Sema3A serum levels in RA patients may reflect its reduced expression in knee joints and/or other organs. Additional investigations to quantify the serum levels of Sema3A from OA and RA patients may contribute to further elucidation of pathogenesis of the disease.

It is unclear which factors regulate Sema3A expression in synovial tissues. However, Fukamachi *et al.* reported that the presence of calcium and histamine could modulate Sema3A expression levels in human keratinocytes and fibroblasts [43]. Since histamine is associated with RA pathogenesis [44,45], it may be responsible for the decreased Sema3A levels observed in RA synovial tissues.

We found that mRNA expression of *VEGF-A* was not significantly altered in OA and RA synovial tissues. Hashimoto *et al.* reported staining intensity for VEGF expression did not differ between RA and OA synovial lining [46] and Lowin *et al.* reported that VEGF_165_ expression did not differ in the chronically inflamed tissue of RA patients and OA patients [47]. In contrast, several reports showed altered expression of VEGF in RA. Lee *et al.* observed significantly higher levels of VEGF protein in RA compared with OA synovial fluid and serum [48]. Kurosawa *et al.* observed significant correlations of serum VEGF levels with DAS28-CRP scores [24]. The elevation of VEGF protein in RA synovial fluid and serum may explain the higher total number of VEGF-producing cells in the region [46]. Indeed, our study showed no signification correlation between *VEGF-A* expression levels and DAS28-CRP scores (Figure 2), but there was a marked increase of synovial tissue thickness of the lining layer in RA (Figure 1). Thus, VEGF levels in RA serum may be increased as previously reported [24]. In an earlier study, Ikeda *et al.* reported *VEGF*_*165 *_was expressed in 41% of RA samples (17 patients) but not in OA samples (8 patients) using reverse transcription-PCR [49]. They also found *NRP1* was up-regulated in RA synovial tissues. However, we did not observe significant alterations of *VEGF-A* (Figure 2B) or *NRP1* (Figure 2C) between OA and RA specimens. Kim *et al.* reported that NRP1 expression was similar in OA and RA using immunohistochemical analyses [50]. These discrepancies might be explained by the difference in sampling numbers, disease duration of the populations studied, extent of inflammation, use of anti-rheumatoid drugs, and/or by different methods for analysis of NRP1 expression levels. Additional investigations in a large sample considering several different conditions are required to clarify these discrepancies.

Although Sema3A inhibits endothelial formation and angiogenesis due to competition with VEGF_165 _[51], this study did not identify a significant correlation between *Sema3A* mRNA expression levels and blood vessel density in RA synovial tissues. This suggests that angiogenesis in RA lesions may be regulated by other mediators, such as placenta growth factor 1, IL-2 and hepatocyte growth factor [47,52].

The imbalance between Sema3A and VEGF may also affect the etiology of RA. Although *VEGF-A* expression levels did not exhibit a significant correlation with DAS28-CRP scores, the *Sema3A/VEGF-A* mRNA ratios demonstrated a relationship with the RA clinical score. Several studies have demonstrated that anti-NRP1 peptides suppressed the survival, adhesion and migration of VEGF_165_-induced synovial cells, which contribute to cartilage destruction in RA [50,53]. VEGF_165_ also increased the production of cytokines by human peripheral blood mononuclear cells [54]. Sema3A may inhibit the action of VEGF_165_ on synovial cells and inflammatory cells in a competitive manner.

Innervation is important in the pathogenesis of arthritis. Primary afferent sensory nerve fibers are proinflammatory, whereas sympathetic nerve fibers are anti-inflammatory [55]. In RA synovial tissues, numbers of sympathetic nerve fibers decreased while sensory nerves increased when compared with OA [56]. The loss of sympathetic nerve fibers in RA may be caused by increased Sema3C and its soluble receptor NRP2 [6,55]. However, the mechanism of increased sensory nerve fibers in RA synovial tissues is not fully understood. Decreased Sema3A expression in RA may facilitate the increase of sensory nerves over sympathetic nerves in inflamed RA synovium [55].

Several limitations of our study should be addressed. First, this study population may be influenced by environmental factors as well as genetic factors. Second, patients enrolled in our study were in the advanced stages of RA disease (mean duration 16.3 years). Thus, our data do not reflect the pathogenesis of RA during the early stages. Third, anti-rheumatoid drugs may alter the expression level of Sema3A and other molecules. In this study 12 patients (40%) received methotrexate (MTX) (mean prednisolone daily dose 2.9 mg), 4 patients (13.3%) received TNF-α blockade (3 patients in combination with MTX, 1 patient in combination with sulfasalazine, mean prednisolone daily dose 3.6 mg), 11 patients received Disease Modifying Antirheumatic Drugs (DMARDs) without MTX (mean prednisolone daily dose 1.6 mg), and 3 patients received prednisolone alone (daily dose 6.2 mg). Although there was no statistically significant difference in *Sema3A* expression, DAS28-CRP, or Rooney’s inflammation score between the MTX–treated group and other groups (data not shown), we cannot rule out the possibility of altered Sema3A expression by anti-rheumatoid drugs, because each group sample size is small in this study.

## Conclusions

This study demonstrated that Sema3A expression levels and the disease activity score in patients with RA negatively correlated with histological parameters in RA synovial tissues. This suggests that Sema3A may have a suppressive effect on the pathological severity of RA. Further elucidation of the role of Sema3A in joints may elucidate the biological and pathological functions of Sema3A and allow the development of new strategies for the treatment of RA.

## Abbreviations

DAS28-CRP: 28-joint disease activity score based on c-reactive protein; HE: Hematoxylin and eosin; IL: Interleukin; NRP1: Neuropilin1; NRP2: Neuropilin2; OA: osteoarthritis; qPCR: Quantitative real-time polymerase chain reaction; RA: Rheumatoid arthritis; Sema3A: Semaphorin3A; Sema3C: Semaphorin3C; Sema3F: Semaphorin3F; Sema3G: Semaphorin3G; SD: Standard deviation; SLE: Systemic lupus erythematosus; TNF-α: Tumor necrosis factor-α; VEGF_165_: Vascular endothelial growth factor 165.

## Competing interests

The authors declare that they have no competing interests. This work was supported by supported by Grants-in-Aid for Scientific Research nos. 30170517 (to T.S) and nos. 23590429 (to Y.N) from the Ministry of Education, Culture, Sports, Science and Technology of Japan (MEXT), and by Grant nos. 07085023 (to Y.G.) from the National Project on Protein Structural and Functional Analyses of MEXT.

## Authors’ contributions

TS participated in the design of the study, performed the experiments and statistical analysis, drafted and revised the manuscript. NF designed the study, analyzed data, drafted and revised the manuscript critically. KK helped with collection and acquisition of the data and the synovium and revised the manuscript critically. NY assisted in scoring histological parameters and revised the manuscript critically. GY designed the study and revised the manuscript critically. ST helped with collection of the clinical materials and revised the manuscript critically. All authors read and approved the final manuscript for publication.

## Pre-publication history

The pre-publication history for this paper can be accessed here:

http://www.biomedcentral.com/1471-2474/14/40/prepub

## Supplementary Material

Additional file 1 **Expression of *VEGF***_***165 ***_**mRNA in synovial tissues: preliminary study.** Expression levels of *VEGF*_*165 *_mRNA were measured by real-time PCR. The mRNA levels were normalized to the expression of *β-actin*. The box plots demonstrate the 10th and 90th percentile (whiskers), the 25th and 75th percentile, and the median. *VEGF*_*165 *_mRNA levels were not significantly altered in RA (n = 25) synovial tissues compared with OA (n = 17) (A). *VEGF*_*165 *_expression levels did not significantly correlate with DAS28-CRP. (B) Click here for file
