# Supplementary figures and images for: Decreased Semaphorin3A expression correlates with disease activity and histological features of rheumatoid arthritis
Source: BMC Musculoskelet Disord. 2013 Jan 23;14:40. doi: 10.1186/1471-2474-14-40 (PMC3558329; doi:10.1186/1471-2474-14-40)

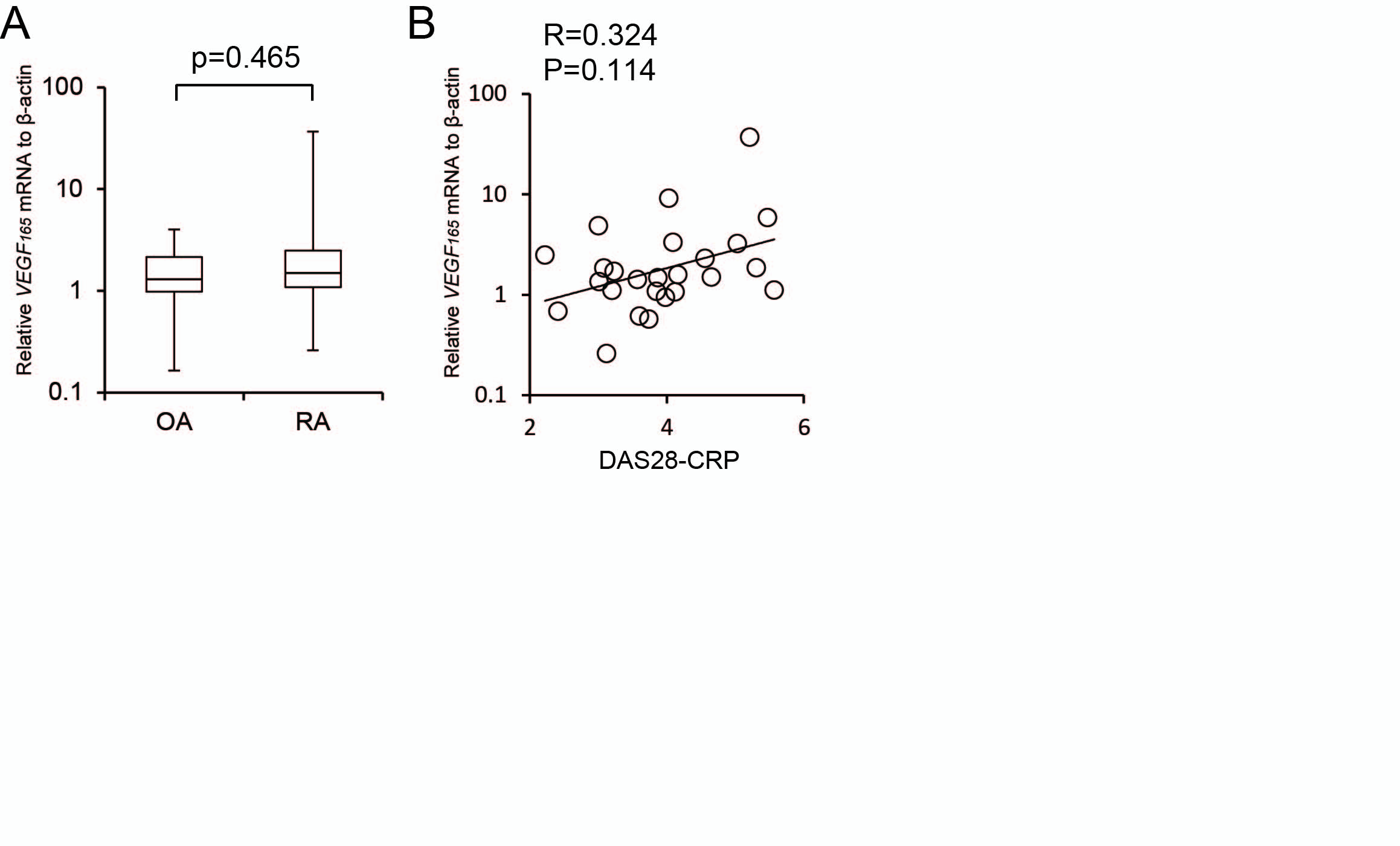

Supplement: Additional file 1 — Expression of VEGF165 mRNA in synovial tissues: preliminary study. Expression levels of VEGF165 mRNA were measured by real-time PCR. The mRNA levels were normalized to the expression of β-actin. The box plots demonstrate the 10th and 90th percentile (whiskers), the 25th and 75th percentile, and the median. VEGF165 mRNA levels were not significantly altered in RA (n = 25) synovial tissues compared with OA (n = 17) (A). VEGF165 expression levels did not significantly correlate with DAS28-CRP. (B) [file 1471-2474-14-40-S1.jpeg]
